# Large bi-axial tensile strain effect in epitaxial BiFeO_3_ film grown on single crystal PrScO_3_

**DOI:** 10.1038/s41598-023-45980-w

**Published:** 2023-11-03

**Authors:** In-Tae Bae, Zachary R. Lingley, Brendan J. Foran, Paul M. Adams, Hanjong Paik

**Affiliations:** 1 Microeletronics Technology Department, The Aerospace Corporation, El Segundo, CA 90009 USA; 2 Materials Processing Department, The Aerospace Corporation, El Segundo, CA 90009 USA; 3 School of Electrical and Computer Engineering, University of Oklahoma, Norman, OK 73019 USA; 4 Center for Quantum Research and Technology, University of Oklahoma, Norman, OK 73019 USA

**Keywords:** Engineering, Materials science

## Abstract

A BiFeO_3_ film is grown epitaxially on a PrScO_3_ single crystal substrate which imparts ~ 1.45% of biaxial tensile strain to BiFeO_3_ resulting from lattice misfit. The biaxial tensile strain effect on BiFeO_3_ is investigated in terms of crystal structure, Poisson ratio, and ferroelectric domain structure. Lattice resolution scanning transmission electron microscopy, precession electron diffraction, and X-ray diffraction results clearly show that in-plane interplanar distance of BiFeO_3_ is the same as that of PrScO_3_ with no sign of misfit dislocations, indicating that the biaxial tensile strain caused by lattice mismatch between BiFeO_3_ and PrScO_3_ are stored as elastic energy within BiFeO_3_ film. Nano-beam electron diffraction patterns compared with structure factor calculation found that the BiFeO_3_ maintains rhombohedral symmetry, i.e., space group of *R3c*. The pattern analysis also revealed two crystallographically distinguishable domains. Their relations with ferroelectric domain structures in terms of size and spontaneous polarization orientations within the domains are further understood using four-dimensional scanning transmission electron microscopy technique.

## Introduction

BiFeO_3_ (BFO) is known as the only multiferroic material that exhibits ferroelectricity and *G*-type antiferromagnetism, simultaneously well above room temperature^1,2^. Thus, BFO has application potential for emerging spintronics technology such as multiple-state memory and magnetic random access memory^3–5^. Early studies on BFO suggested that ferroelectric response in BFO was weak, i.e., 3.5 µC cm^−2^, in addition to the weak magnetic response, i.e., antiferromagnetism^1,6,7^_._ Thus, this material drew little attention. However, pioneering works in early 2000s demonstrated that its ferroelectric response is almost an order of magnitude higher than the previously reported value by showing that the spontaneous polarization value of BFO is ~ 60 µC cm^−2^. This spontaneous polarization value has been reported for epitaxial BFO film^8^ and high quality single crystalline bulk BFO^9,10^. In addition, with the availability of high quality single crystalline oxide substrates with lattice parameters and crystal structure that are similar to BFO, significantly increased spontaneous polarization values of 90–115 µC cm^−2^ were reported for epitaxially grown BFO films^11–14^. It is worth noting that the epitaxial BFO films are under biaxial strain caused by the difference in crystal symmetry and/or lattice parameter of the oxide substrate on which it grows. Furthermore, the biaxial strain caused by single crystal substrates with similar lattice parameter can be stored as *elastic* strain within BFO film rather than relaxed through lattice defect formations such as misfit dislocations. This is attributed to the structurally flexible nature of BFO that can accommodate many percent of elastic strain^15,16^. Since the elastic strain stored within BFO thin films modifies its lattice parameter and potentially crystal symmetry leading to changes in spontaneous polarization and magnetic property, considerable experimental and theoretical efforts have been devoted to understand the elastic strain effects on the crystal symmetry changes within epitaxial BFO films^11,17–20^. As a result, a variety of metastable BFO phases, such as rhombohedral^6^ tetragonal-like^15,16,21–25^, orthorhombic^26^, orthorhombic-like monoclinic^27^, monoclinic^24,28–32^, and triclinic^33,34^, are found depending on the substrate type and surface orientation of the substrate used. Interestingly, while extensive studies have already been performed to investigate the compressive strain effect on epitaxial BFO film, few studies have been performed for the tensile strain effect^32,35^ presumably owing to the limited availability of high quality perovskite-based oxide substrates with the lattice parameters larger than that of BFO^36,37^.

It is also worth noting that while most of the experimental works have used X-ray diffraction (XRD) techniques combined with pseudocubic notation-based BFO unit cell to investigate the misfit strain induced *unit cell distortion*, no discussion has been made on how the misfit strain affects *basis location* in the unit cell, which is another important factor to properly evaluate a crystal structure. In order to address this issue, we have proposed to use hexagonal notation based BFO unit cell^27,38–42^. Since hexagonal notation describes rhombohedral unit cell of *unstrained* BFO impeccably, it has proven highly effective to accurately evaluate: (1) overall crystal symmetry and (2) the amount of misfit strain stored elastically within strain engineered BFO films. In addition, when structure factor calculations using hexagonal notation are compared with wide range reciprocal space information, such as nano-beam electron diffraction and/or X-ray reciprocal space mapping, it allows us to evaluate the crystal structure of elastically strained BFO with no ambiguity^27,35,38,39^.

In this study, biaxial tensile strain in an epitaxial BFO film grown on a PrScO_3_ (PSO) single crystal using molecular beam epitaxy is investigated as follows:

1. Elastic strain induced BFO crystal structure is evaluated using aberration (*C*_*s*_)-corrected scanning transmission electron microscopy (STEM) with nano-beam electron diffraction (NBED) and structure factor calculation.
2. Quantitative strain measurement within the epitaxial BFO film is performed using precession electron diffraction (PED) in tandem with XRD and X-ray reciprocal space mapping.
3. Ferroelectric domain structure and spontaneous polarization orientation within the domains are examined using four dimensional STEM differential phase contrast (4D STEM DPC) technique.

## Results and discussion

**Figure 1:**
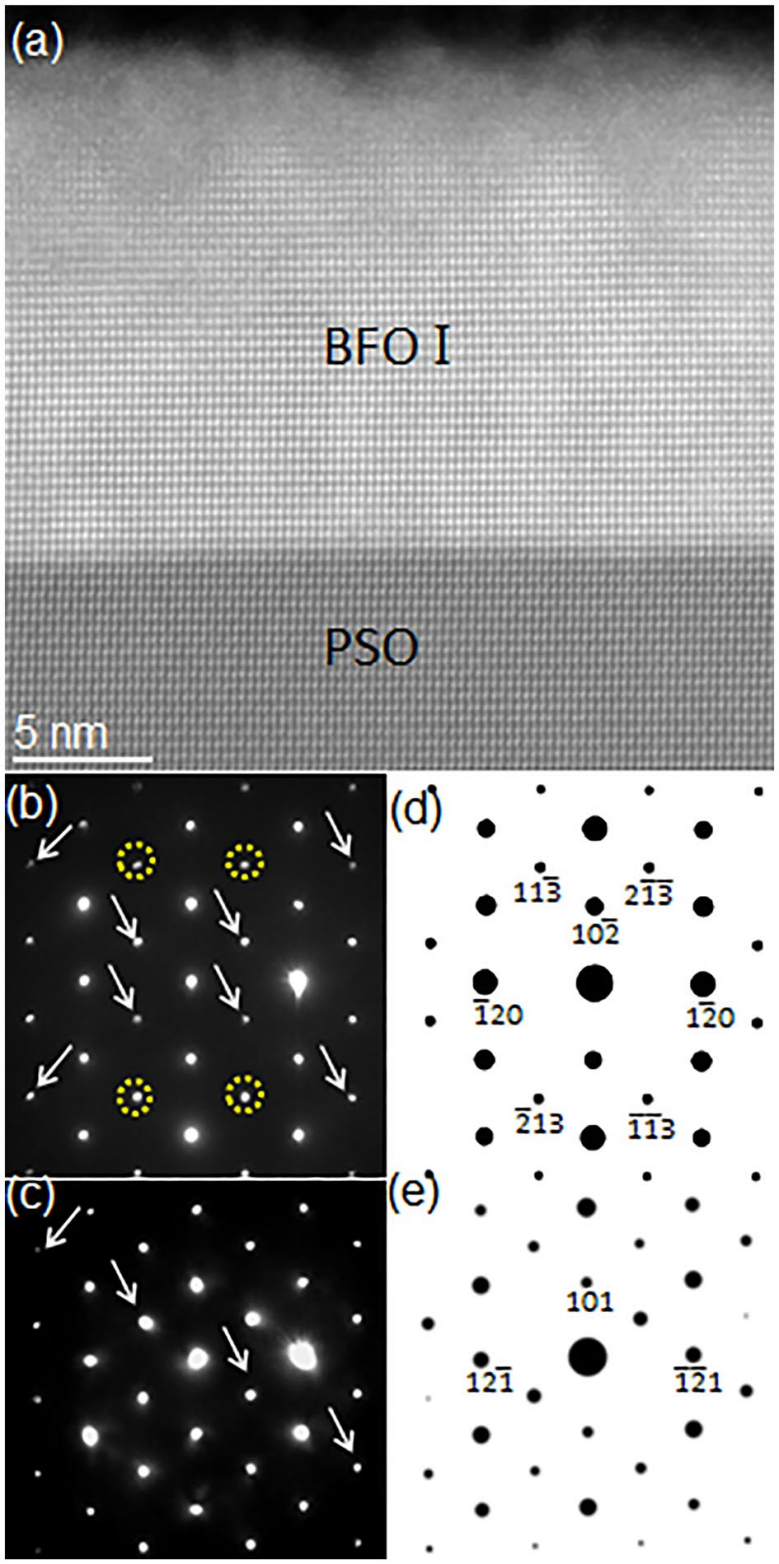
(**a**) A cross-sectional HAADF-STEM image of epitaxial BFO grown on PSO along $[\overline{1 }11]$_o_ zone axis with NBED patterns from (**b**) BFO I and (**c**) PSO together with their corresponding structure factor calculations in (**d**) and (**e**), respectively. (**d**) Confirms that (**b**) corresponds to [211]_h_ rhombohedral BFO.

Figure 1a shows a high angle annular dark field (HAADF)-STEM image of the BFO films grown on (101)_o_ PSO substrate (space group: *Pnma*, *a* = 0.5780 nm, *b* = 0.8025 nm, *c* = 0.5608 nm, *α* = *β* = *γ* = 90°) along $[\overline{1 }11]$_o_ zone axis (Subscript “o” denotes orthorhombic notation)^43^. It exhibits ~ 20 nm BFO grown on PSO substrate. Note that the BFO film shows up brighter than PSO substrate because Bi atom within BFO, which is heavier than Pr and Sc in PSO, provides more signals to the HAADF detector located at the collection semi-angle of 80–100 mrad^44^.

To acquire information on the crystal structure of BFO and the epitaxial relation between BFO and PSO, nano-beam electron diffraction (NBED) patterns were recorded from BFO and PSO with a probe size of ~ 10 nm as shown in Fig. 1b,c. For accurate BFO phase identification in terms of: (1) lattice vector sizes and (2) basis locations in unit cell, structure factor, *F*_*hkl*_, where *hkl* represents a specific Bragg’s reflection, was calculated for all BFO phases discussed in previous reports (including theoretically predicted metastable ones) that provide all the necessary crystallographic information, such as space group, lattice parameter, and basis locations in unit cell^20,21,27,45,46^. These BFO phases are summarized in Table 1.

**Table 1:** Summary of BFO phases that were previously reported with all the crystallographic information that includes lattice parameter, space group and basis location.

Symmetry	Space group	Lattice parameter	Technique used	Year
Rhombohedral^44^	*R3c*	*a* = 0.5678 nm, *c* = 1.3982 nm, *a* = *b* = 90°, *g* = 120°	Neutron diffraction	2007
Monoclinic^45^	*P2*_*1*_*/m*	*a* = 0.5615 nm, *b* = 0.7973 nm, *c* = 0.5647 nm, *a* = 90°, *b* = 90°, *g* = 90.1°	X-ray diffraction	2008
Tetragonal^21^	*P4mm*	*a* = 0.367 nm, *c* = 0.464 nm, *a* = *b* = *g* = 90°	First-principles	2006
Monoclinic^20^	*Pc*	*a* = 0.7291 nm, *b* = 0.5291 nm, *c* = 0.5315 nm, *a* = 90°, *b* = 139.46°, *g* = 90°	First-principles	2011
Monoclinic^20^	*Cm*	*a* = 0.9354 nm, *b* = 0.7380 nm, *c* = 0.3804 nm, *a* = 90°, *b* = 86.60°, *g* = 90°	First-principles	2011
Orthorhombic^20^	*Pna2*_*1*_	*a* = b = 0.5314 nm, *c* = 0.9452 nm, *a* = *b* = *g* = 90°	First-principles	2011
Monoclinic^20^	*Cc*	*a* = 1.0604 nm, *b* = 0.5322 nm, *c* = 0.5323 nm, *a* = 90°, *b* = 62.80°, *g* = 90°	First-principles	2011
Orthorhombic^20^	*Pnma*	*a* = 0.5650 nm, b = 0.7770 nm, *c* = 0.5421 nm, *a* = *b* = *g* = 90°	First-principles	2011
Orthorhombic^20^	*Pna2*_*1*_	*a* = 0.5702 nm, b = 0.5507 nm, *c* = 0.8036 nm, *a* = *b* = *g* = 90°	First-principles	2011
Orthorhombic-like monoclinic^27^	*Cm*	*a* = 0.9262 nm, *b* = 0.7582 nm, *c* = 0.3791 nm, *a* = *g* = 90°, *b* = ~ 90°	Electron diffraction & First-principles	2017

Also calculated was the structure factor of PSO substrate^43^ to investigate the epitaxial relation with BFO overlayer. The electron diffraction calculation was based on kinematical approximation:

$$F_{hkl} = \sum\limits_{n} {f_{n} \exp } \left[ {2\pi i\left( {hx_{n} + ky_{n} + lz_{n} } \right)} \right],$$

where *f*_*n*_ is the atomic scattering factor for atom *n* at fractional coordinates (*x*_*n*_,*y*_*n*_,*z*_*n*_). By comparing NBED patterns to the structure factor calculation results from all the BFO phases mentioned above, the NBED pattern from BFO layer shown in Fig. 1b is identified as the [211]_h_ zone axis of rhombohedral BFO, i.e., bulk BFO crystal structure^45^, as shown in the corresponding structure factor calculation of Fig. 1c. Note that (1) the [211]_h_ orientation of rhombohedral BFO is based on three-index *hexagonal* notation (Subscript h denotes hexagonal notation) rather than pseudocubic (pc) notation that disregards: (1) ~ 0.55° of rhombohedral distortion and (2) basis locations in unit cell. However, since pc notation has been more widely used, we have summarized the relation between the hexagonal notations used in the current study and the corresponding pc notations in Table S1. The diffraction spots denoted by white arrows in Fig. 1b result from double diffraction, i.e., dynamical scattering effect, as discussed previously^27,35,38,39^. The Bragg’s reflections denoted with yellow circles are indexed, for examples, $\left(11\overline{3 }\right)$_h_, $\left(2\overline{13 }\right)$_h_, $\left(\overline{11 }3\right)$_h_ and $\left(\overline{2 }13\right)$_h_ as shown in Fig. 1d (see Table S1 for the corresponding pc notations). Note that these are termed rhombohedral BFO signature reflections since none of the BFO phases (reported experimentally or theoretically) but rhombohedral BFO can produce them as discussed previously^27,38–40,42^. Since one of the critical structural characteristics of rhombohedral BFO is oxygen octahedral rotation, it is worth investigating whether oxygen octahedral rotation is preserved in this epitaxial BFO. Figure S1 show atomic models of (a) pseudocubic-approximated, i.e., perovskite (space group: $Pm\overline{3 }m$; lattice parameter *a* = 0.396 nm) and (b) rhombohedral (space group: *R3c*), BFOs viewed along equivalent orientation, i.e., [110]_pc_ for Fig. S1a, and [110]_pc_, i.e., [211]_h_, for Fig. S1), respectively. While no oxygen octahedral rotation shows in Fig. S1a, it is clearly visible with oxygen atoms run zig-zag horizontally in Fig. S1b. The corresponding structure factor calculations are shown in Figs. S1a’,b’, respectively. Note that while Fig. S1a’ exhibits only fundamental reflections, such as [001]_pc_, [$\overline{1 }10$]_pc_ and [$\overline{1 }11$]_pc_, extra reflections denoted by arrows show up together with the fundamental reflections in Fig. S1b’. This indicates that oxygen octahedral rotation in rhombohedral BFO causes extra reflections seen in diffraction patterns. Since the extra Bragg’s reflections are clearly found in Fig. 1b, oxygen octahedral rotation is thought to be preserved in the current epitaxial BFO.

Note that rhombohedral symmetry found here is different than monoclinic symmetry found in an epitaxial BFO film grown on PSO substrate by Chen et al*.*^32^ While they used X-ray reciprocal space mapping for out-of-plane and in-plane Bragg’s peaks to accurately measure the slight shifts in Bragg’s peak locations from those in bulk BFO, the symmetry of basis atom locations of the monoclinic phase was not provided in their study. Thus, it is hard to directly compare their study with the current study. Meanwhile, a more recent study on an epitaxial BFO film grown on PSO substrate using quantitative STEM technique concluded that the BFO film preserves rhombohedral-like feature, which agrees with the current study^47^. For convenience, let us call [211]_h_ BFO domain “BFO I”. The NBED pattern from PSO in Fig. 1c matches the $[\overline{1 }11]$_o_ zone axis of PSO with (101)_o_ reflection along surface normal orientation (see Fig. 1e) as expected. The reflections resulting from dynamical scattering effect are denoted with white arrows in Fig. 1c^48^. Based on the results from Fig. 1d,e, the epitaxial relation between BFO I and PSO is as follows:

[211]_h_ of BFO I // $[\overline{1 }11]$_o_ of PSO ; $\left(10\overline{2 }\right)$_h_ of BFO I // (101)_o_ of PSO.

**Figure 2:**
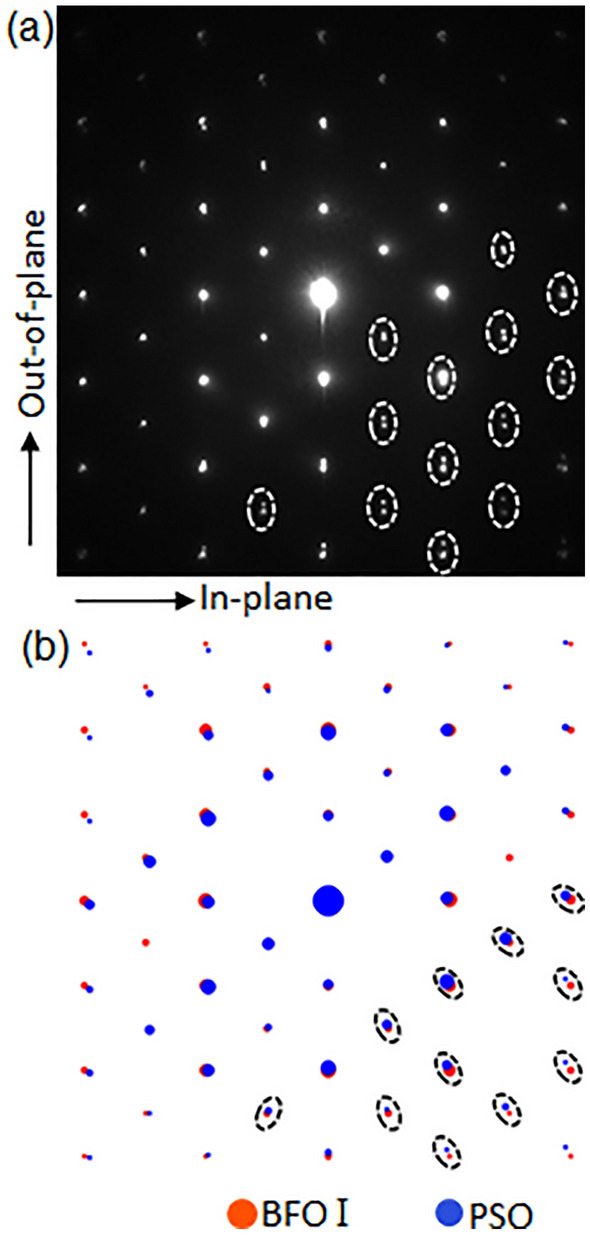
(**a**) A NBED pattern acquired from an area including BFO I and PSO substrate shown in Fig. 1(a, b) structure factor calculation result using unstrained BFO and unstrained PSO. The Bragg’s reflections split vertically along out-of-plane orientation in (**a**), whereas those split radially in (**b**), indicating the in-plane components of BFO I scattering vectors are the same as that of PSO.

Figure 2 shows an NBED pattern obtained from an area (using ~ 10 nm probe) that includes both BFO and PSO to identify whether the lattice misfit with PSO is stored as elastic strain in BFO layer or not. It can be readily noticed that the Bragg’s reflections (denoted by white ellipses) from BFO and PSO split along out-of-plane orientations in Fig. 2a. Note that the split should occur radially if BFO is under no elastic strain as predicted by Fig. 2b that shows a structure factor calculation using *unstrained* BFO and *unstrained* PSO. In addition, the split of Bragg’s reflections along out-of-plane orientation in Fig. 2a indicates the scattering vector components within BFO and PSO are the same along in-plane orientation. This means that the interplanar distances of BFO and PSO are the same along in-planed orientation. While the equilibrium, i.e., *unstrained*, interplanar distance of BFO along in-plane orientation, i.e., BFO($1\overline{2 }0$)_h_, is 0.279 nm^45^, the measured one is the same as interplanar distance of PSO along in-plane orientation, i.e., PSO($\overline{12 }1$)_o_ = 0.284 nm^43^. Since the measured interplanar distance of BFO($1\overline{2 }0$)_h_, i.e., 0.284 nm, is larger than unstrained one, i.e., 0.279 nm, BFO I is under tensile strain along in-plane orientation which is denoted with an arrow at the bottom-left in Fig. 2a.

**Figure 3:**
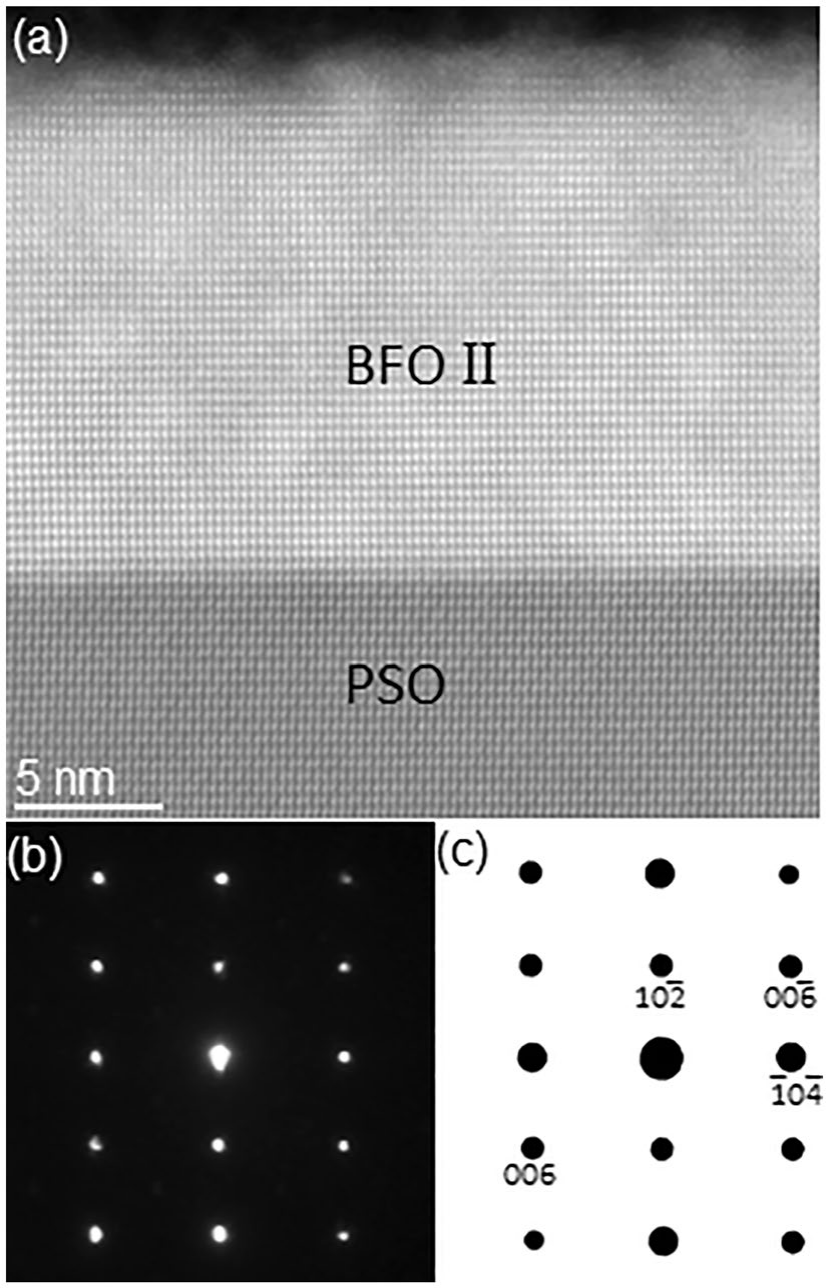
(**a**) A cross-sectional HAADF-STEM image from an area adjacent to Fig. 1a with (**b**) a NBED pattern from BFO II and (**c**) its corresponding structure factor calculation. (**c**) confirms that (**b**) corresponds to $[0\overline{1 }0$]_h_ rhombohedral BFO.

Figure 3a is a HAADF-STEM image of an adjacent area. While the contrast in BFO film appears similar with that in Fig. 1a, the NBED pattern from the area (see Fig. 3b) is distinctively different from Fig. 1b. Structure factor calculation revealed that Fig. 3b corresponds to [$0\overline{1 }0$]_h_ zone axis of rhombohedral BFO as shown in Fig. 3c^45^. Since this BFO domain has a different zone axis than BFO I shown in Fig. 1a, it is referred to as “BFO II”. Based on the results from Figs. 1e and 3c, the epitaxial relation between BFO II and PSO is as follows:

[$0\overline{1 }0$]_h_ of BFO II // $[\overline{1 }11]$_o_ of PSO; $\left(10\overline{2 }\right)$_h_ of BFO II // (101)_o_ of PSO.

**Figure 4:**
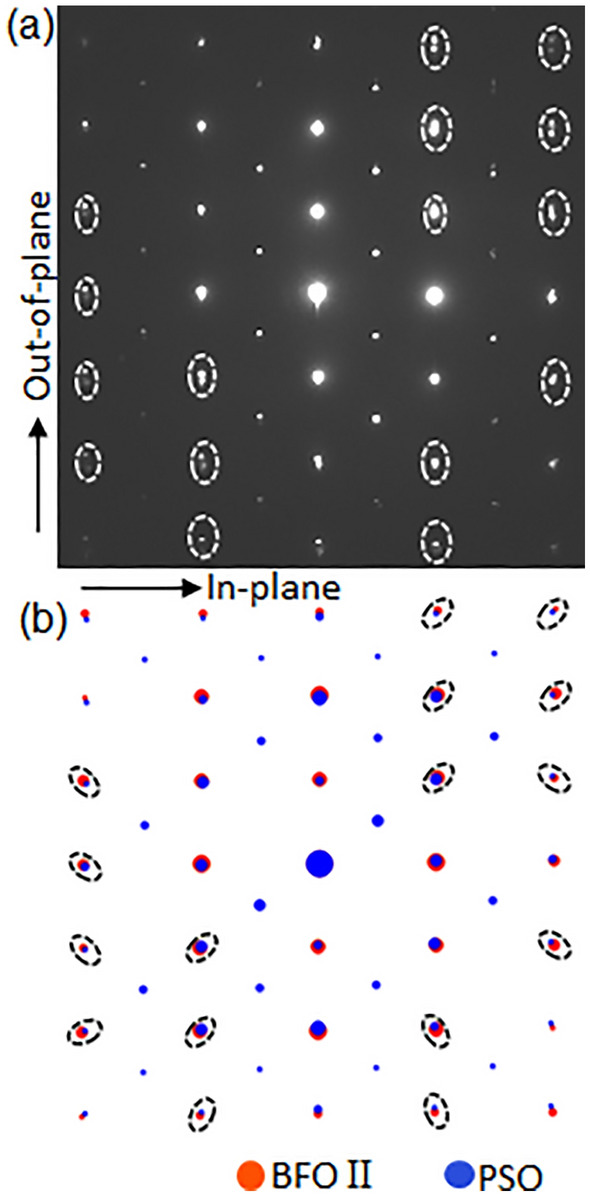
(**a**) A NBED pattern acquired from an area including BFO II and PSO substrate shown in Fig. 3a, and (**b**) structure factor calculation result using unstrained BFO and unstrained PSO. The Bragg’s reflections split vertically along out-of-plane orientation in (**a**) whereas those split radially in (**b**), indicating the in-plane components of BFO II scattering vectors are the same as that of PSO.

Figure 4a presents NBED patterns from both BFO II and PSO. The vertical split of BFO and PSO Bragg’s reflections (denoted by white ellipses) is different from the radially oriented split (as denoted by black ellipses in Fig. 4b) that should occur between *unstrained* BFO and *unstrained* PSO. This indicates the scattering vectors of BFO II and PSO are the same along in-plane orientation, which is the same characteristic found between BFO I and PSO (see Fig. 2b). While the interplanar distance of *unstrained* BFO along in-plane orientation, i.e., BFO$(\overline{1 }0\overline{4 })$_h_ is 0.281 nm, the measured one is the same as interplanar distance of PSO along in-plane orientation, i.e., PSO($\overline{12 }1$)_o_ = 0.284 nm^45^, indicating that BFO II is under tensile strain for the same reason as for BFO I. It is worth noting that while the difference of *unstrained* in-plane lattices, i.e., ($1\overline{2 }0$)_h_ = 0.279 nm for BFO I and ($\overline{1 }0\overline{4 }$)_h_ = 0.281 nm for BFO II can be distinguished based on the difference in NBED patterns shown in Figs. 1b and 3b, pc notations of the corresponding in-plane lattices for BFO I and BFO II are ($\overline{1 }10$)_pc_ = 0.280 nm, and ($\overline{11 }0$)_pc_ = 0.280 nm, respectively. This indicates pc notation cannot distinguish the difference.

The NBED data (showing that the interplanar distance of BFO is the same as that of PSO along the in-plane orientation) is further verified by X-ray reciprocal space mapping data as exhibited in Fig. S2. It provides a reciprocal space map from the vicinity of PSO($2\overline{2 }4$)_o_ and BFO($4\overline{26 }$)_h_, i.e., BFO($\overline{1 }13$)_pc_, peaks. Note that Q_z_ and Q_x_ are along PSO[101]_o_, i.e., out-of-plane, and PSO[$\overline{12 }1$]_o_, in-plane, orientations, respectively. It is readily noticed that the in-plane components of scattering vectors, i.e., Q_x_/2π, read 3.519 nm^−1^ for PSO($2\overline{2 }4$)_o_ and 3.5191 nm^−1^ for BFO($4\overline{26 }$)_h_, i.e., BFO($\overline{1 }13$)_pc_. This indicates the in-plane scattering components of BFO is the same as that of PSO, which is in good agreement with the NBED results shown in Figs. 2 and 4.

**Figure 5:**
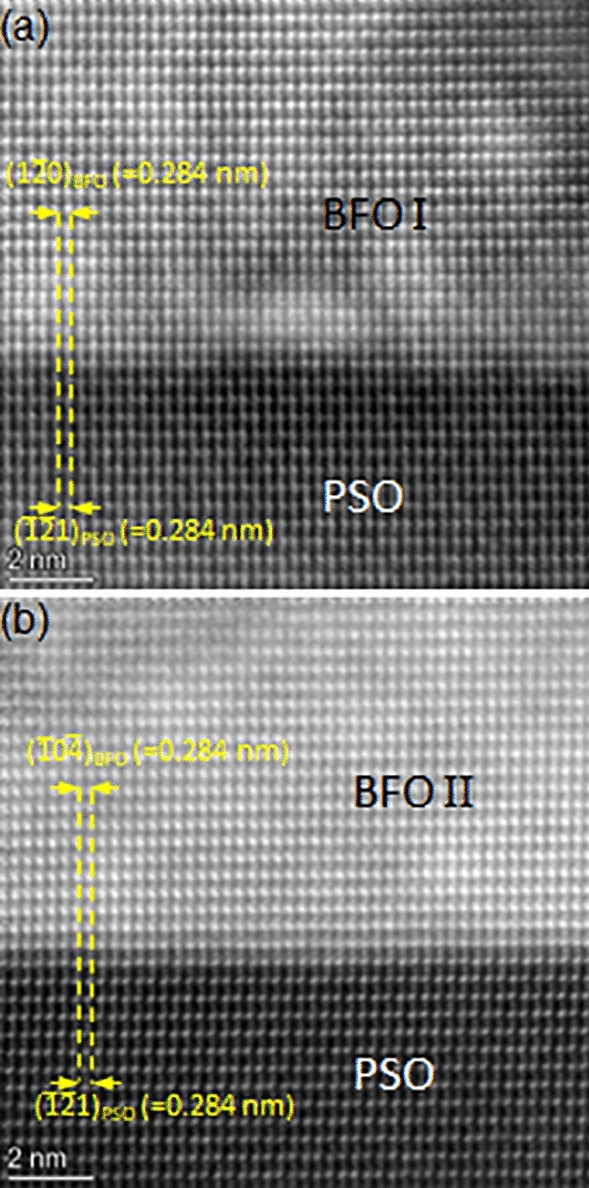
Lattice resolution HAADF-STEM images from (**a**) BFO I/PSO and (**b**) BFO II/PSO interfaces along PSO$[\overline{1 }11]$_o_ zone axis. The interplanar distances of BFO I and BFO II along in-plane orientation are the same as those of PSO with no signs of misfit dislocations at the interfaces.

In order to provide a direct evidence of lattice misfit causing elastic strain within BFO, atomic resolution HAADF-STEM technique was employed as shown in Fig. 5. It can be readily noticed that PSO($\overline{12 }1$)_o_ lattice plane is commensurate with BFO lattice planes across BFO/PSO interface for both BFO I i.e., Fig. 5a, and BFO II, i.e., Fig. 5b. Besides, no misfit dislocations are found in BFO I and BFO II at the interfaces. Misfit dislocations often showing up as threading dislocations are known to play a role to relax elastic strain when dislocation density is higher than the threshold value (e.g., ~ 10^11^ cm^−2^ for (Ba_x_Sr_1–x_)TiO_3_ film)^49,50^. Thus, it can be concluded that the epitaxial BFO in the present work is under *elastic* tensile strain caused by lattice misfit with PSO.

**Figure 6:**
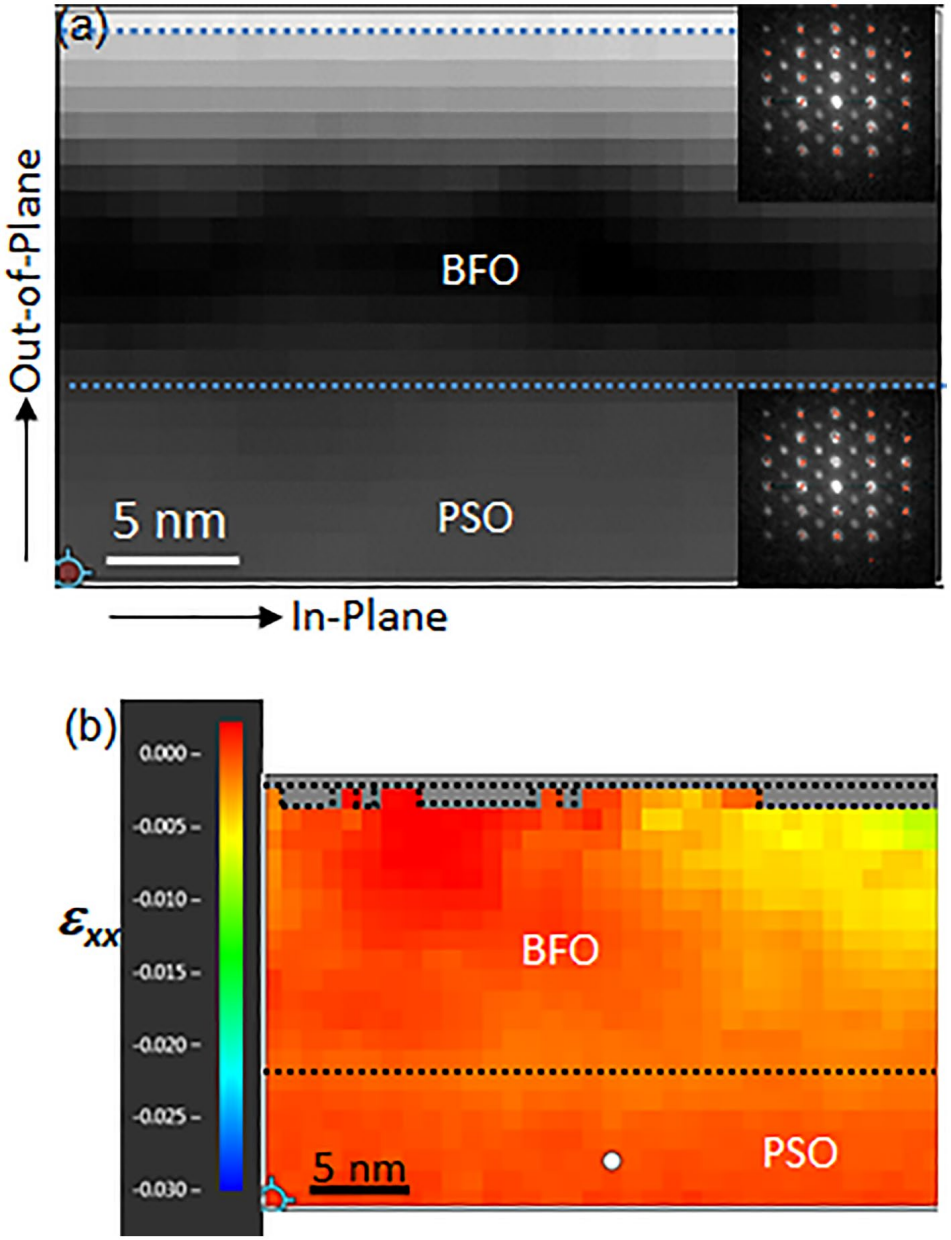
(**a**) A virtual BF image acquired from PED along PSO$[\overline{1 }11]$_o_ zone axis (with PED pattern examples from BFO and PSO shown as insets at top-right for BFO and at bottom-right for PSO), and (**b**) *ε*_*xx*_ extracted from PED patterns.

The status of biaxial tensile strain across the entire BFO film is further investigated using precession electron diffraction technique (PED) with 120 keV electron and a precession angle of ~ 0.8°. In Fig. 6a is shown a virtual bright-field (BF) image was obtained using the direct beam, i.e., (000) central spot in diffraction pattern, from each PED pattern (see the insets at top right for BFO and bottom right for PSO as examples) as electron probe scans through the area. The interplanar distance along in-plane orientation at each pixel in Fig. 6a is calculated using high index Bragg’s reflections as shown in the red cross hairs along red horizontal lines in the insets for high accuracy measurement. Figure 6b exhibits the ratio of interplanar distance difference along in-plane orientation, ${\varepsilon }_{xx}=\frac{{d}_{BFO (in-plane)}-{d}_{PSO (in-plane)}}{{d}_{PSO (in-plane)}}$. It is readily noticed that the contrast in BFO is even throughout the entire area except for the top right area with yellow, i.e., *ε*_*xx*_ =  ~ 0.006, and green, i.e., *ε*_*xx*_ =  ~ 0.008, shades. This may have to do with sample warpage or slight relaxation happening at the area. Note that some of the PED patterns from the area near top surface of BFO is excluded as they include the noise from neighboring amorphous Pt film that was deposited for FIB sample preparation (see the grayed-out area near the top surface of BFO enclosed with dotted lines in Fig. 6b). The mean and standard deviation of *ε*_*xx*_ from the entire area are -0.0009 and 0.0017, respectively. This confirms in-plane interplanar distance, i.e., $\left(1\overline{2 }0\right)$_h_, within BFO film is virtually the same as that in PSO, i.e., 0.284 nm, throughout the entire BFO area. This is in good agreement with NBED (see Figs. 2, 4) and lattice resolution HAADF-STEM (Fig. 5) results. Since the equilibrium interplanar distances of BFO I$\left(1\overline{2 }0\right)$_h_ and BFO II$(\overline{1 }0\overline{4 })$_h_ along in-plane orientation are 0.279 nm and 0.281 nm^45^, it becomes clear that BFO layer is under tensile strain, i.e. ~ 1.79% for BFO I and ~ 1.06% for BFO II. Thus, the averaged tensile strain between BFO I and BFO II is ~ 1.43% along [211]_h_, i.e., [110]_pc_, orientation.

**Figure 7:**
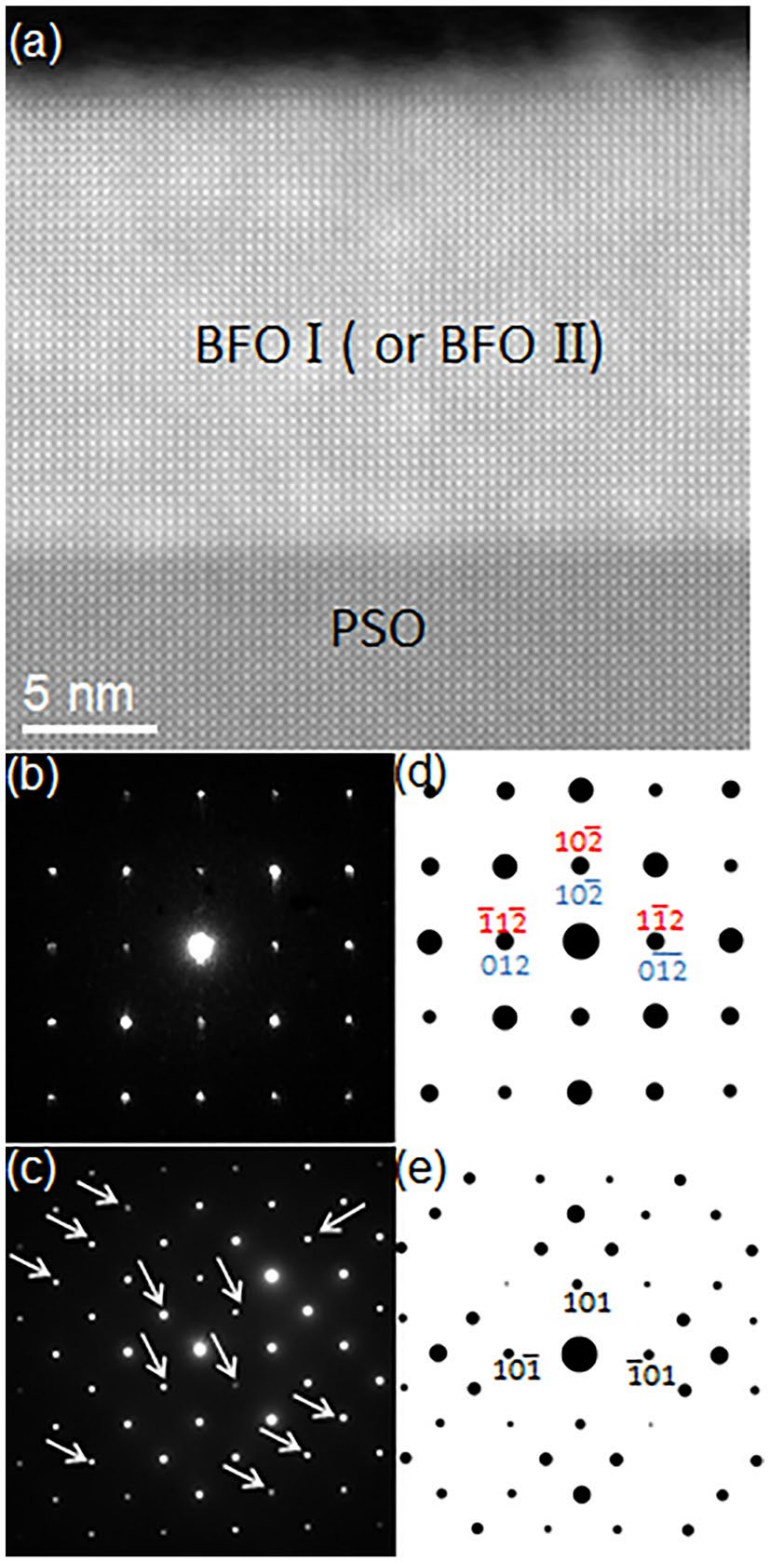
(**a**) A cross-sectional HAADF-STEM image of epitaxial BFO grown on PSO along $[010]$_o_ zone axis with NBED patterns from (**b**) BFO and (**c**) PSO together with their corresponding structure factor calculations in (**d**) and (**e**), respectively. (**d**) Confirms that (**b**) corresponds to either [241]_h_, i.e., BFO I, or $[2\overline{2 }1]$_h_, i.e., BFO II, of rhombohedral BFO.

**Figure 8:**
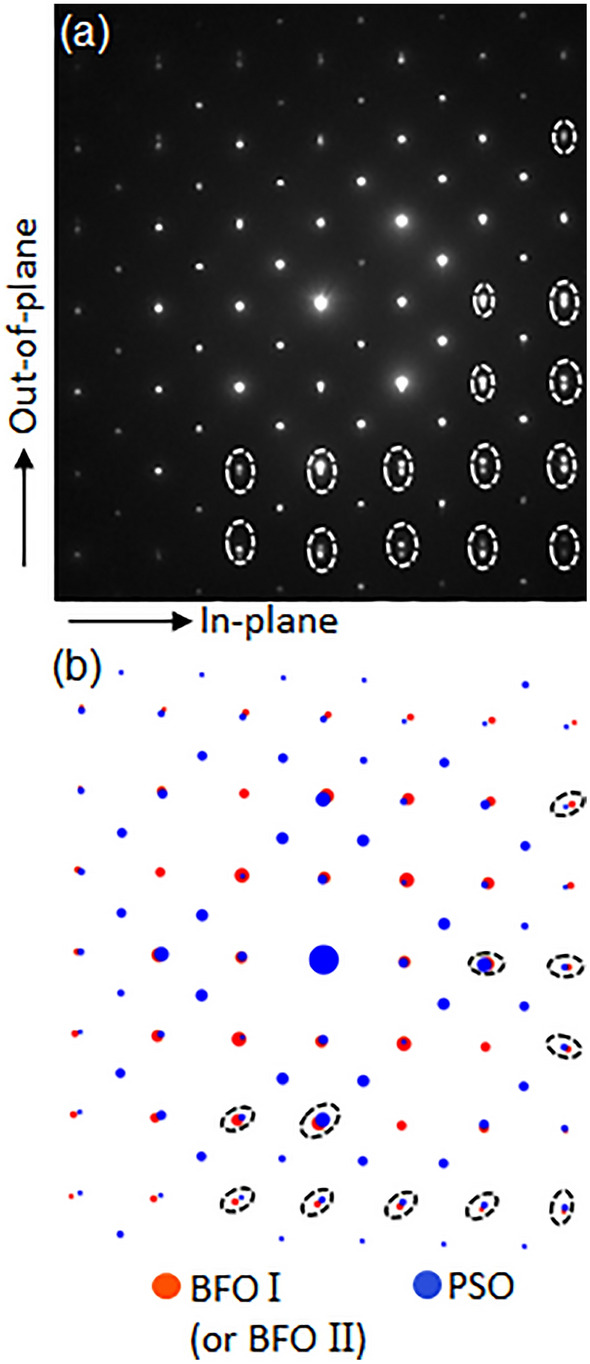
(**a**) A NBED pattern acquired from an area including BFO and PSO substrate shown in Fig. 7a, and (**b**) structure factor calculation result using unstrained BFO and unstrained PSO. The Bragg’s reflections split vertically along out-of-plane orientation in (**a**) whereas those split radially in (**b**), indicating the in-plane components of BFO scattering vectors are the same as that of PSO.

**Figure 9:**
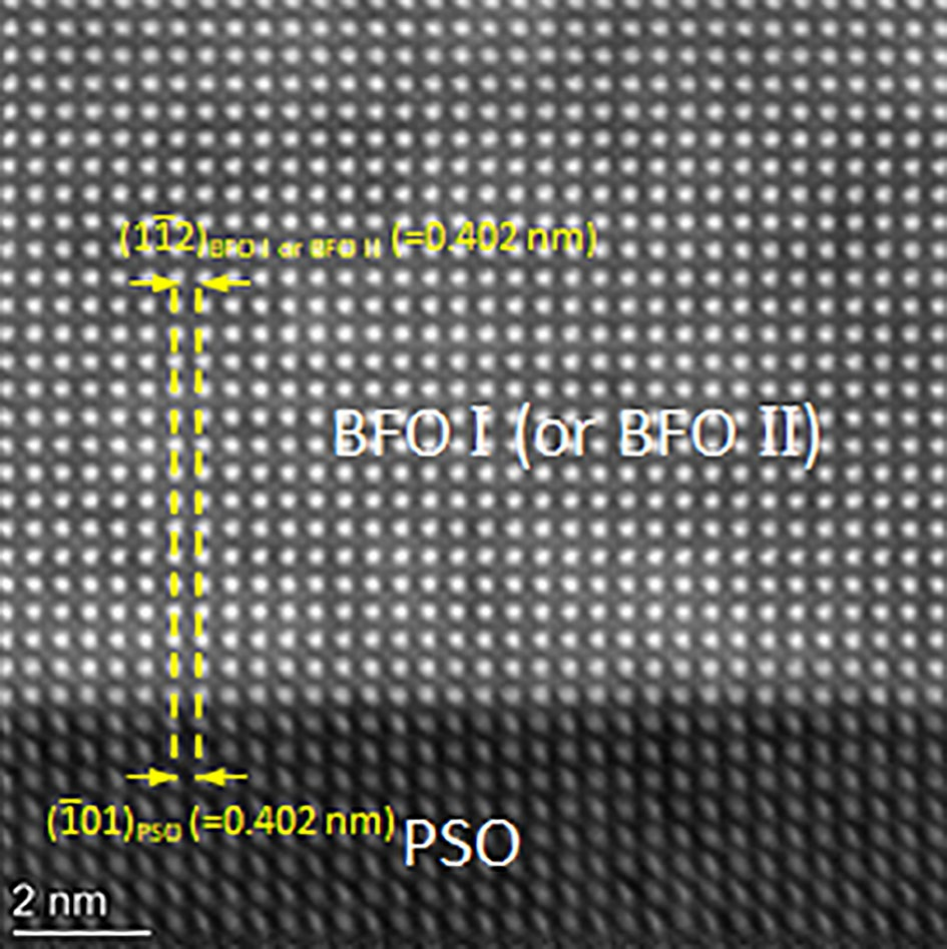
Atomic resolution HAADF-STEM images at BFO/PSO interface along PSO$[010]$_o_ zone axis. The interplanar distances of BFO along in-plane orientation are the same as that of PSO with no signs of misfit dislocations at the interface. The BFO areas shown here could be either BFO I or BFO II as discussed for Fig. 7b,d.

**Figure 10:**
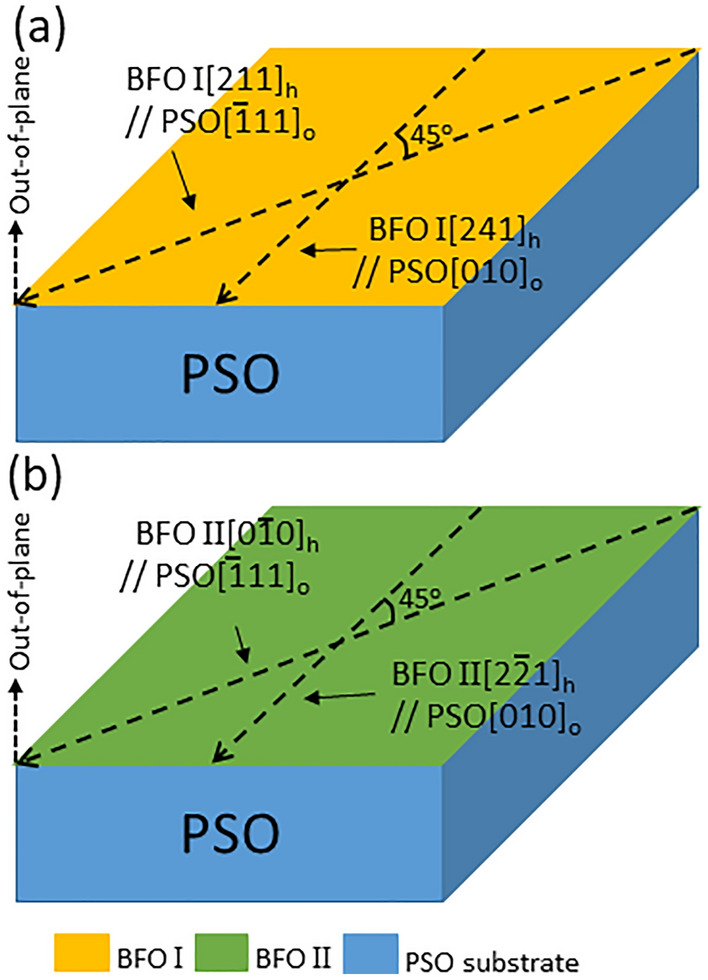
A schematic showing epitaxial relations: (**a**) between BFO I and PSO, and (**b**) BFO II and PSO. BFO I and BFO II are ~ 90° away around out-of-plane orientation.

Another cross-sectional TEM sample is prepared along PSO[010]_o_, a zone axis which is 45° away from PSO$[\overline{1 }11]$_o_ around the out-of-plane orientation to confirm the elastic strain status within BFO film as shown in Fig. 7. Figure 7a is a HAADF-STEM image showing ~ 20 nm thick BFO epitaxial layer grown on PSO. NBED patterns acquired from BFO and PSO are shown in Fig. 7b,c. It is worth noting that the NBED pattern in Fig. 7b can be interpreted either [241]_h_ (see the indices in red) or $[2\overline{2 }1]$_h_ (see the indices in blue) of rhombohedral BFO as shown in Fig. 7d. Since BFO[241]_h_ and BFO$[2\overline{2 }1]$_h_ are ~ 45° away from BFO[211]_h_, i.e., BFO I, and BFO$[0\overline{1 }0]$_h_, i.e., BFO II, respectively, Fig. 7b corresponds to either BFO I or BFO II. Figure 7c matches the structure factor calculation of PSO$[\overline{1 }11]$_o_ as shown in Fig. 7e. The Bragg’s reflections caused by dynamical scattering are denoted with white arrows in Fig. 7c^48^. An NBED pattern using ~ 10 nm probe that spanned the BFO/PSO interface is acquired to investigate elastic strain status within BFO layer as shown in Fig. 8. The Bragg’s reflections aligned along out-of-plane orientation is readily found (see the white ellipses in Fig. 8a). This is the same characteristic, i.e., elastic tensile strain, as in Figs. 2 and 4, indicating that the interplanar distance of BFO is the same as that of PSO along in-plane orientation. This can be confirmed with an atomic resolution HAADF-STEM image in Fig. 9. It shows that PSO in-plane lattice, i.e., PSO($\overline{1 }01)$_o_, is commensurate with BFO in-plane lattice, i.e., $\left(1\overline{1 }2\right)$_h_ of either BFO I or BFO II, with no signs of misfit dislocation. Note that while the measured interplanar distance of $\left(1\overline{1 }2\right)$_h_ of either BFO I or BFO II is 0.402 nm, i.e., the same as that of PSO($\overline{1 }01)$_o_, in equilibrium it is 0.396 nm^43^. Thus, it is shown that BFO layer is under ~ 1.51% tensile strain along PSO[010]_o_ orientation. The anisotropic elastic strain found here is in good agreement with the coherent tensile strain found along two in-plane orientations within 13 nm-thick epitaxial BFO film grown on PSO substrate previously^32^. Based on the NBED analysis results in Figs. 2, 4 and 8, a schematic is drawn in Fig. 10 to visualize the epitaxial relations between BFO I and PSO (see Fig. 10a) and between BFO II and PSO (see Fig. 10b). It is also worth noting that BFO I and BFO II are 90° apart around the $\left(10\overline{2 }\right)$_h_ plane normal, i.e., out-of-plane, orientation as discussed elsewhere^42^.

**Figure 11:**
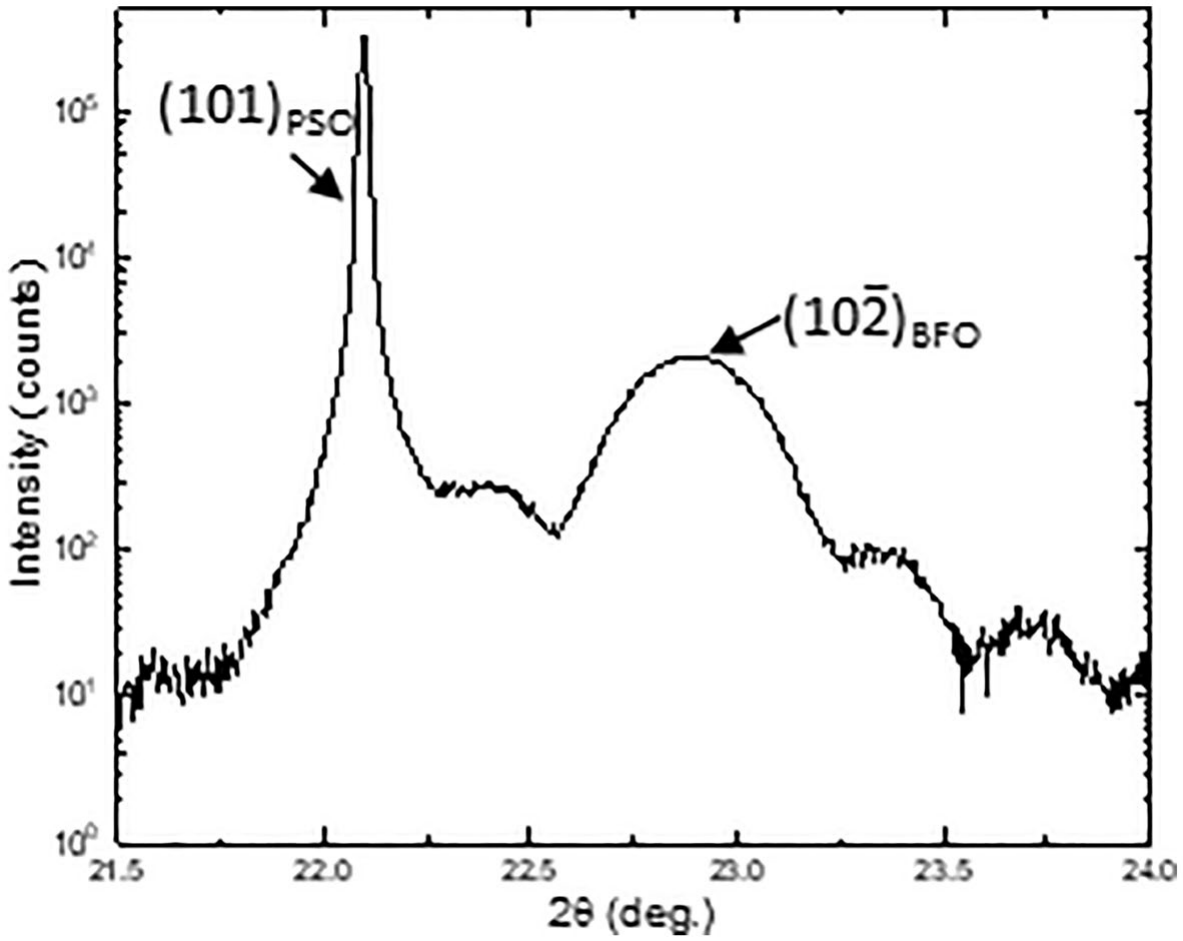
A XRD measurement with θ–2θ geometry, i.e., along out-of-plane orientation. A peak at 22.89° is from BFO$\left(10\overline{2 }\right)$_h_. Interplanar distance corresponding to 22.89° is ~ 0.388 nm which is smaller than that of *unstrained* BFO$\left(10\overline{2 }\right)$_h_, ~ 0.396 nm, indicating BFO film is under uniaxial compressive strain along out-of-plane orientation.

Now let’s turn our attention to interplanar distance within BFO along out-of-plane orientation. Note that both BFO I and BFO II have $\left(10\overline{2 }\right)$_h_ plane along out-of-plane orientation as shown in Figs. 1 and 3. To precisely measure the volume averaged interplanar distance along out-of-plane orientation, X-ray diffraction technique is used with Cu Kα radiation, hybrid optics with Ge monochromator. In Fig. 11 is shown X-ray intensity as a function of 2θ acquired using θ–2θ geometry, i.e., out-of-plane orientation. A sharp peak located at 22.09° corresponds to PSO(101)_o_ = 0.402 nm, whereas a broad peak at 22.89° is from BFO$\left(10\overline{2 }\right)$_h_. Besides, thickness fringes are seen as well. The full width half maximum of BFO$\left(10\overline{2 }\right)$_h_ is measured 0.254° which is much larger than 0.019° of PSO(101)_o_ owing to peak broadening resulting from ~ 20 nm thickness of BFO film. The measured interplanar distance of BFO$\left(10\overline{2 }\right)$_o_ based on 2θ = 22.89° turns out ~ 0.388 nm. Given the *unstrained* interplanar distance of BFO$\left(10\overline{2 }\right)$_h_ is 0.396 nm, BFO film is under a uniaxial compressive strain (~ 2.02%) along out-of-plane orientation as the result of biaxial tensile strain along in-plane orientation. The uniaxial compressive strain of ~ 2.02% along out-of-plane orientation is larger than ~ 1.45% in-plane biaxial tensile strain averaged between PSO[010]_o_,i.e., BFO[100]_pc_, and PSO$[\overline{1 }11]$_o_ i.e., BFO[110]_pc_ orientations. This is presumably due to the fact that while biaxial tensile strain is exerted two dimensionally across the entire BFO film, the tensile strain is uniaxial, i.e. one dimensional. Since two-dimensional tensile strain effect along in-plane orientation shows up one dimensional compressive strain along out-of-plane orientation, the out-of-plane compressive strain (~ 2.02%) turns out larger than in-plane tensile strain (~ 1.45%). This is in good agreement with the results on BFO film grown on KTaO_3_ substrate previously^35^.

By using the in-plane and out-of-plane strains, Poisson’ ratio (ν), can be acquired as follows:

ν = ε_xx_/ε_zz_/(ε_zz_/ε_xx_ – 2), where ε_xx_ and ε_zz_ are in-plane and out-of-plane lattice mismatches, respectively^31,51^. ν for the current study turns out ~ 0.41. This is comparable to ν =  ~ 0.49 reported previously using epitaxial BFO films grown on various oxide substrates^31^. This confirms the fact that BFO film possesses higher Poisson’s ratio than other perovskite-based materials. The reason could be associated with structural flexibility within perovskite-based BFO crystal structure that has already been discussed in terms of: (1) multiple metastable phases available^20,21^ and (2) small perovskite tolerance factor (~ 0.88) allowing for large degrees of rotation and/or tilting of oxygen octahedra^31^.

**Figure 12:**
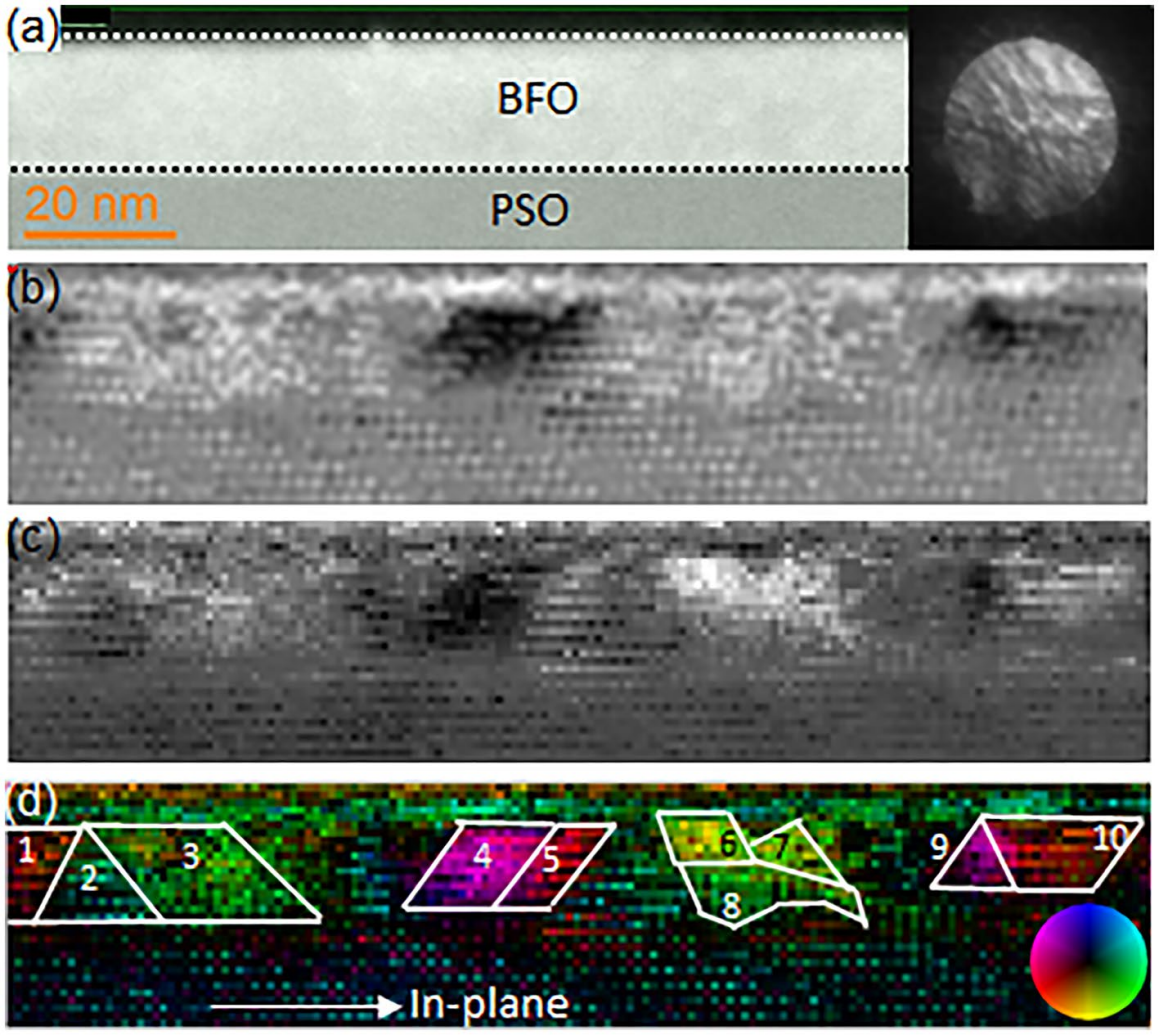
(**a**) A cross-sectional HAADF-STEM image of epitaxial BFO grown on PSO along $[\overline{1 }11]$_o_ zone axis with an example of direct beam, i.e., central disk in diffraction pattern, as an inset the right corner. Direct beam shifts, caused by the electric fields resulting from spontaneous polarization in dielectric domains are shown in terms of two orthogonal orientations, i.e., (**b**) *dx* and (**c**) *dy*. (**d**) Vector displacement map with a color wheel as an inset bottom right corner. Ferroelectric domains are denoted by white lines with numbers.

To further investigate ferroelectric domain structures and their relations with the crystallographically distinguished domain structures, i.e., BFO I and BFO II, 4D STEM DPC is performed along PSO$[\overline{1 }11]$_o_ zone axis as shown in Fig. 12. 4D STEM DPC technique collects a diffraction pattern at each pixel in a HAADF-STEM image (see Fig. 12a) and measures the displacement of the direct beam, i.e., central disk, in each diffraction pattern. An example of direct beam recorded on a pixelated detector is shown an inset at the right corner in Fig. 12a. Note that the radius of the direct beam is 25 mrad. Then, the direct beam displacement, which occurs when incident electrons are deflected by the electric field due to spontaneous polarization within BFO ferroelectric domain^52^, are calculated along two orthogonal orientations, i.e., *dx* (see Fig. 12b) and *dy* (see Fig. 12b) by center of mass approach^53^. The color-coded vector displacement map is calculated using *dx* and *dy* data as shown in Fig. 12d where the intensity scales to the magnitude of the vector field and the color represents its orientation as shown by the color wheel at bottom-right corner. While no clear contrast of ferroelectric domains is exhibited in a HAADF-STEM image obtained along PSO$[\overline{1 }11]$_o_ zone axis as shown in Fig. 12a, the vector displacement map in Fig. 12d clearly shows ferroelectric domains size of 10–20 nm. The polarization orientations of the ferroelectric domains (denoted 1–10) and their relations with crystallographic domains, i.e., BFO I and BFO II, are summarized in Table 2.

**Table 2:** Summary of the spontaneous polarization angles, i.e., mean and standard deviation, with respect to in-plane orientation for each ferroelectric domains denoted 1–10 in Fig. 12d.

Domain	Angle with respect to in-plane orientation (deg.)	
	Mean	SD
1 (BFO II)	142.0	8.4
2 (BFO II)	− 19.8	16.3
3 (BFO II)	25.7	8.6
4 (BFO II)	− 155.6	2.9
5 (BFO II)	161.6	8.0
6 (BFO I)	89.7	7.4
7 (BFO I)	83.1	11.2
8 (BFO II)	26.5	6.5
9 (BFO II)	− 158.6	4.8
10 (BFO II)	137.1	7.4

Note that the mean and standard deviation are based on 10 pixels from the central area of each ferroelectric domains denoted 1–10 in Fig. 12d. Their relations with crystallographic domains, i.e., BFO I and BFO II are also provided.

Note that (1) the angles are measured with respect to in-plane orientation denoted in Fig. 12d, and (2) the angles acquired from 10 pixels at the center of each domain were used to derive mean and standard deviation. It is worth noting that the domains with polarization angles 89.7° and 83.1° correspond to BFO I whereas the others correspond to BFO II. This has to do with the fact that while polarization orientation of BFO II has both of in-plane and out-of-plane components viewed along BFO[110]_pc_//PSO$[\overline{1 }11]$_o_ zone axis [see the location of 006_h_, i.e., 111_pc_, reflection along which spontaneous polarization occurs in Fig. 3c], that of BFO I has out-of-plane components only. This is because the BFO I is 90° off BFO II around out-of-plane orientation as mentioned earlier (see Fig. 10)^42^. Han et al.^54^ recently investigated spontaneous polarization orientations in epitaxial BFO grown on PSO(001)_o_ substrate along [110]_pc_ orientation using aberration corrected TEM to identify that, while 71° domain wall consists of polarization components of both in-plane and out-of-plane, 180° domain wall consists of out-of-plane component only. Thus, their result is consistent with the current study. In addition, the mean polarization angles of BFO II found here seem to be smaller with respect to in-plane orientation (except for domains 1 and 10), than the theoretical angle of 35.6°, as discussed elsewhere^42^. Although the standard deviations for the mean polarization angles in BFO I and BFO II are rather high, this might indicate the polarization orientations found here under biaxial tensile strain have more in-plane orientation component than that in unstrained BFO. It was reported that biaxial tensile strain could cause BFO polarization orientation to rotate within (110)_pc_ plane towards in-plane orientation^32,55^. Note that (110)_pc_ corresponds to the cross-section plane of BFO in Fig. 12 of which zone axis is [211]_h_. Thus, the polarization orientations rotation towards in-plane orientation found for BFO I and BFO II seems to agree with those previous reports.

The polarization, i.e., ferroelectric, orientations summarized in Table 2 show four polarization, i.e., ferroelectric, variants (for example, see domains 1, 2, 3 and 4 whose angles with respect to the in-plane orientation denoted in Fig. 12d are 142.0°, − 19.8°, 15.7°, and − 155.6°, respectively). Thus, the result found here is different from two ferroelectric variants found in BFO grown on DyScO_3_ (DSO) substrate, but consistent with the four ferroelectric variants found in BFO grown on STO buffered DSO substrate^56^. It is interesting to note that while 71° ferroelectric domain structure is dominantly found in the BFO grown on DSO substrate using PFM and X-ray reciprocal space mapping technique^56^, the current study with BFO grown on PSO substrate found ferroelectric domain structures with angles other than the typical 71°, 109°, and 180° using 4D STEM DPC technique. For example, the angle between domains 1 and 4 is ~ 62°, whereas that between domains 2 and 3 is ~ 46° (see Table 2). This is the result of polarization orientation being rotated toward in-plane orientation owing to the tensile strain applied in the BFO as discussed earlier. It is unclear how the rotation of polarization orientation found in the current study would affect the ferroelectric domain structure and ferroelectric variants in BFO. Further study is needed to clarify this point.

## Summary

In summary, large biaxial tensile strain is investigated in an epitaxially grown BFO film on single crystalline PSO substrate in terms of BFO crystal symmetry, and ferroelectric domain structure using advanced STEM techniques. NBED patterns in comparison with structure factor calculations, along two zone axes showed no evidence of breakdown of rhombohedral symmetry, i.e., space group of *R3c*. However, lattice resolution STEM images, PED and XRD confirm that the misfit strain caused by PSO substrate is stored as elastic strain, i.e., ~ 1.45% biaxial tensile strain along in-plane orientation accompanied by ~ 2.2% uniaxial compressive strain along out-of-plane orientation, in the epitaxially grown BFO film. This results in a Poisson’s ratio of ~ 0.41. In addition, two crystallogrphically distinguished BFO domains, i.e., BFO I and BFO II, are confirmed by NBED analysis. Their relations with ferroelectric domains are further studied using 4D STEM DPC technique. It visualizes: (1) ferroelectric domain sizes (10–20 nm) and (2) polarization orientation with the ferroelectric domains that have four ferroelectric variants. By combining polarization and the crystallographic orientation information, it is revealed that the ferroelectric domains showing only out-of-orientation component corresponds to BFO I, whereas those possessing both in-plane and out-of-plane components are BFO II. Larger in-plane polarization component found in BFO II than the theoretical one seems to agree with the tensile strain induced polarization rotation within BFO discussed in previous reports.

## Methods

An epitaxial BFO film was grown on a (101)_o_ PSO substrate using molecular beam epitaxy in PARADIM facility at Cornell University. Cross-sectional TEM samples were prepared using Thermo Fisher Helios 600 dual beam focused ion beam. Ga ion beam was gradually decreased from 30 to 2 kV to minimize ion beam induced damage. For HAADF-STEM, NBED and 4D STEM DPC, a Thermo Fisher Titan Themis G2 300 equipped with a probe corrector was used with a convergence semi-angle of 25 mrad, and 770 mm camera length. To minimize knock-on damage from incident electron to BFO film, 120 keV high tension was used to ensure that the maximum energy transfers to Bi, Fe, and O are below a typical threshold displacement energy of ~ 25 eV^57^. A Gatan OneView™ CMOS camera with readout binned to 512 × 512 pixels was used to collect diffraction data for 4D STEM DPC. Gatan Microscopy Suite software was used to analyze the 4D STEM DPC data. NBED patterns were calibrated using $\left(10\overline{2 }\right)$ and $\left(\overline{1 }20\right)$ reflections from unstrained PSO substrate. For XRD analysis, Panalytical X’pert Pro with Cu Kα radiation was used in tandem with a hybrid optics equipped with Ge monochromator to measure out-of-plane Bragg’s peak with high accuracy. X-ray reciprocal space mapping was performed using Rigaku SmartLab SE equipped with Cu Kα radiation, Ge(220) two bounce monochromator, and HyPix-400 2D detector.

### Supplementary Information

Supplementary Information.

## Data Availability

All data generated or analyzed during this study are included in the published article. In case, one wished to consult further information, it will be made available through the corresponding author on reasonable request.
