# Supplementary material for: Large bi-axial tensile strain effect in epitaxial BiFeO3 film grown on single crystal PrScO3
Source: Sci Rep. 2023 Nov 3;13:19018. doi: 10.1038/s41598-023-45980-w (PMC10624869; doi:10.1038/s41598-023-45980-w)
Supplement: Supplementary file 1 — Supplementary Information. [file 41598_2023_45980_MOESM1_ESM.docx]

Large bi-axial tensile strain effect in epitaxial BiFeO_3_ film grown on single crystal PrScO_3_

In-Tae Bae^1,^*, Zachary R. Lingley^1^, Brendan J. Foran^1^, Paul M. Adams^2^, & Hanjong Paik^3^

^1^Microeletronics Technology Department, The Aerospace Corporation, California 90009, USA. ^2^Materials Processing Department, The Aerospace Corporation, California 90009, USA. ^3^School of Electrical and Computer Engineering, Center for Quantum Research and Technology, University of Oklahoma, Norman, Oklahoma 73019, USA. *Correspondence and requests for materials should be addressed to I.T.B. (e-mail: [intae.bae@aero.org](mailto:intae.bae@aero.org))

Supplementary Information


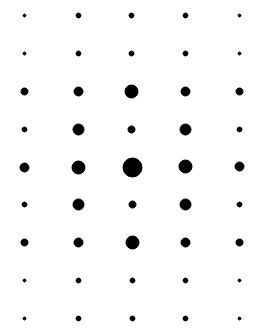

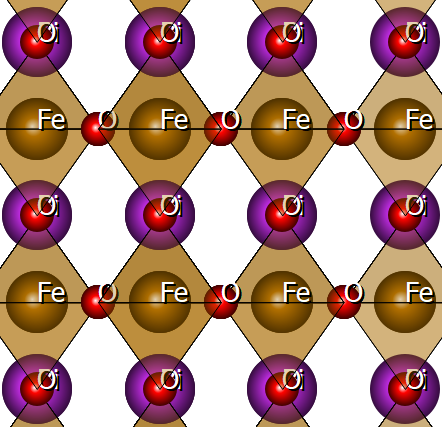

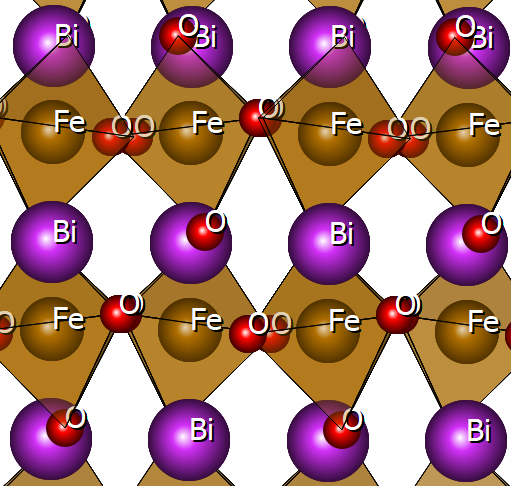

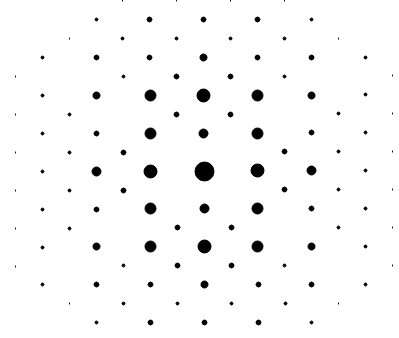


113_pc_

**-**

112_pc_

**-**

111_pc_

**-00**

**-**

001_pc_

110_pc_

003_pc_

111_pc_

**-**

001_pc_

110_pc_

**-**

(a)

(a’)

002_pc_

(b’)

(b)

Supplementary Figure S1. Atomic models of (a) pseudocubic-approximated, i.e., perovskite (space group: $Pm\bar{3}m$; lattice parameter *a* = 0.396 nm) and (b) rhombohedral (space group: *R3c*), BFOs viewed along equivalent orientation, i.e., [110]_pc_ for (a), and [110]_pc_, i.e., [211]_h_ for (b), respectively. While oxygen octahedral rotation is not visible in (a), that is clearly visible with oxygen atoms running zig-zag horizontally in (b). The corresponding structure factor calculations are shown (a’) and (b’), respectively. While (a’) exhibits fundamental reflections only, (b’) shows not only fundamental reflections but also extra columns of reflections (denoted by arrows) which are caused by the oxygen octahedral rotation in rhombohedral BFO unit cell.

Supplementary Figures


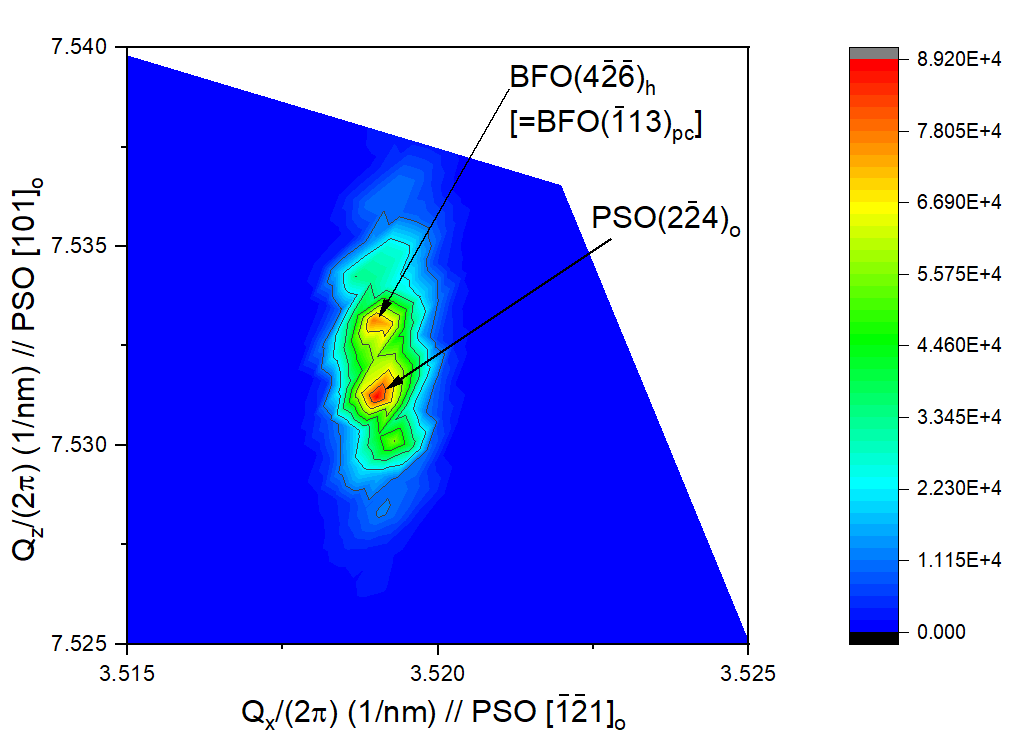


Supplementary Figure S2. X-ray reciprocal space mapping showing the vicinity of PSO($2\bar{2}4$)_o_ and BFO($4\bar{26}$)_h_, i.e., BFO($\bar{1}13$)_pc_, reflections. Note that the two peaks line up vertically along PSO[101]_o_, i.e, out-of-plane orientation, indicating that the in-plane components of the scattering vectors of the two peaks are identical.

Supplementary Tables

| Hexagonal BFO | Pseudocubic BFO | Corresponding orthorhombic PSO orientation |
| --- | --- | --- |
| [241]_h_ | [100]_pc_ | [010]_o_ |
| [211]_h_ | [110]_pc_ | $[\bar{1}11]$_o_ |
| $[0\bar{1}0$]_h_ | [$\bar{1}10$]_pc_ | $[\bar{11}1]$_o_ |
| [001]_h_ | [111]_pc_ | [$\bar{1}10$]_o_ |
| $\left( 11\bar{3} \right)$_h_ | (0.5 -0.5 1.5)_pc_ | $\left( 211 \right)$_o_ |
| $\left( 2\bar{13} \right)$_h_ | (-0.5 0.5 1.5)_pc_ | ($1\bar{1}2$)_o_ |
| $\left( \bar{11}3 \right)$_h_ | (-0.5 0.5 -1.5)_pc_ | $\left( \bar{211} \right)$_o_ |
| $\left( \bar{2}13 \right)$_h_ | (0.5 -0.5 -1.5)_pc_ | ($\bar{1}1\bar{2}$)_o_ |
| ($1\bar{2}0$)_h_ | ($\bar{1}10$)_pc_ | $\left( \bar{12}1 \right)$_o_ |
| ($\bar{1}0\bar{4}$)_h_ | ($\bar{11}0$)_pc_ | $\left( 1\bar{21} \right)$_o_ |

Supplementary Table S1. Hexagonal notation conversion to pseudocubic notation. PSO orientations and planes corresponding to those of BFO are also shown.
